# The Effect of High-Altitude on Human Skeletal Muscle Energetics: ^31^P-MRS Results from the Caudwell Xtreme Everest Expedition

**DOI:** 10.1371/journal.pone.0010681

**Published:** 2010-05-19

**Authors:** Lindsay M. Edwards, Andrew J. Murray, Damian J. Tyler, Graham J. Kemp, Cameron J. Holloway, Peter A. Robbins, Stefan Neubauer, Denny Levett, Hugh E. Montgomery, Mike P. Grocott, Kieran Clarke, Caudwell Xtreme Everest Research Group

**Affiliations:** 1 Department of Physiology, Anatomy and Genetics, University of Oxford, Oxford, Oxfordshire, United Kingdom; 2 The Oxford Centre for Clinical Magnetic Resonance Research, John Radcliffe Hospital, Oxford, Oxfordshire, United Kingdom; 3 School of Clinical Sciences, University of Liverpool, Liverpool, Merseyside, United Kingdom; 4 Centre for Altitude, Space, and Extreme Environment Medicine, University College London, London, United Kingdom; 5 Institute for Human Health and Performance, University College London, London, United Kingdom; Pennington Biomedical Research Center, United States of America

## Abstract

Many disease states are associated with regional or systemic hypoxia. The study of healthy individuals exposed to high-altitude hypoxia offers a way to explore hypoxic adaptation without the confounding effects of disease and therapeutic interventions. Using ^31^P magnetic resonance spectroscopy and imaging, we investigated skeletal muscle energetics and morphology after exposure to hypobaric hypoxia in seven altitude-naïve subjects (trekkers) and seven experienced climbers. The trekkers ascended to 5300 m while the climbers ascended above 7950 m. Before the study, climbers had better mitochondrial function (evidenced by shorter phosphocreatine recovery halftime) than trekkers: 16±1 vs. 22±2 s (mean ± SE, *p*<0.01). Climbers had higher resting [Pi] than trekkers before the expedition and resting [Pi] was raised across both groups on their return (PRE: 2.6±0.2 vs. POST: 3.0±0.2 mM, *p*<0.05). There was significant muscle atrophy post-CXE (PRE: 4.7±0.2 vs. POST: 4.5±0.2 cm^2^, *p*<0.05), yet exercising metabolites were unchanged. These results suggest that, in response to high altitude hypoxia, skeletal muscle function is maintained in humans, despite significant atrophy.

## Introduction

The metabolic response to impaired regional (e.g. vascular disease) or systemic (e.g. cardiopulmonary disease) oxygen delivery is hard to determine, given the confounding effects of the disease state itself and of therapeutic interventions. The study of healthy individuals exposed to altitude-related (hypobaric) hypoxia offers an approach to this problem. As yet, the pattern of response remains unclear: mitochondrial density, far from increasing [1], [2], may in fact fall [3] (albeit in the context of muscle atrophy [4]), while enzymes used in anaerobic glycolysis may be upregulated in the muscle of high altitude natives and acclimatised lowlanders [5], [6] (a suggestion supported by *in vitro* studies [7]). Hypoxic cachexia, although well described, remains poorly understood.

There are several hypoxia-tolerance strategies known in other mammals. For example, sea turtles reduce cellular oxygen consumption during hypoxia by prioritizing certain ATP-consuming processes at the expense of others [8]. Whether this might also be a feature of human physiology is not known. In addition, the oxygen-cost of ATP rephosphorylation might be reduced, although the notion that oxygen efficiency might be modulated in humans in response to hypoxia remains deeply controversial [9], [10].

Phosphorus magnetic resonance spectroscopy (^31^P-MRS) and magnetic resonance imaging (MRI) allow the non-invasive assessment of mitochondrial function, muscle cross-sectional area and phosphate metabolism in humans. We thus applied these techniques to the prospective study of two groups of healthy individuals exposed to sustained hypobaric hypoxia.

## Methods

### Ethics statement

The protocol was approved by the University College (University of London) Ethical Committee. Written informed consent was obtained from all participants.

### Subjects and experimental outline

We studied fourteen healthy men aged 24–48 years from the Caudwell Xtreme Everest expedition (CXE) [11]. Of these, seven were altitude-naïve (‘trekkers’) and seven were experienced climbers who had previously climbed above 6800 m without incident (‘climbers’). Subjects underwent health screening to ensure that they were fit both to complete an ascent to high altitude and to take part in the planned programme of research activity. Prior to departure, all subjects attended the Oxford Centre for Clinical Magnetic Resonance Research (OCMR). On the morning after an overnight fast, their heights and weights were recorded, and body mass index (BMI) calculated.

Each subject performed a series of plantar flexion exercises in the bore of a Siemens Trio 3T clinical magnetic resonance system, with a dual-tuned ^31^P and ^1^H surface coil placed under the widest part of the right gastrocnemius. The exercise protocol was: 5 minutes rest, 5 minutes very light exercise (warm-up), 7 minutes recovery, 5 minutes at 4 W (EX1), 7 minutes recovery (REC1), 5 minutes at 5 W (EX2), 5 minutes recovery (REC2). The exercise work rates were established in pilot experiments, and were optimized to substantially deplete muscle phosphocreatine while remaining sustainable throughout the exercise periods. Muscle cross-sectional area, phosphorus metabolites, phosphocreatine (PCr) recovery halftime and intracellular pH were calculated as described below.

All the subjects ascended from Oxford to Everest Base Camp over approximately 14 days. The trekkers then descended to sea-level via Kathmandu (1300 m) over 7 days and were retested within 48 hours. All of the climbers ascended to at least the South Col (7950 m), and four successfully summitted (8848 m). The climbers then descended to sea-level over 10–17 days and were retested approximately a week after leaving Kathmandu. Twelve subjects (7 trekkers and 5 climbers) returned to OCMR following CXE.

### Nuclear Magnetic Resonance Protocols

A series of gradient-echo ‘scout’ images were acquired in the transverse, sagittal and coronal planes to ensure correct positioning of the leg, and to allow for localized shimming of the region of interest (ROI). Image parameters were as follows: FOV 250×250 mm, matrix size 256×128, 3 slices per orientation, slice thickness 8 mm, TR/TE 15/5 ms, excitation flip angle 40° and bandwidth 180 Hz/Px. This was immediately followed by localized shimming of the ROI using the phasemap algorithm as implemented on the 3T system. This increased the local magnet homogeneity and reduced the linewidths in the acquired spectra.

Prior to the acquisition of the ^31^P spectra during the leg exercise protocol, three baseline scans were acquired to allow calculation of correction factors for partial saturation, caused by both the short repetition time (TR) used in the spectral acquisition, and nuclear Overhauser enhancement (NOE). The first baseline scan was acquired with a long TR to ensure complete relaxation of all resonances. The scan parameters were as follows: TR 30 s, TE 0.35 ms, bandwidth 2000 Hz, 8 averages, 512 acquired data points, excitation flip angle 90° and no NOE pulses. The second baseline scan was run with TR 500 ms, TE 0.35 ms, bandwidth 2000 Hz, 60 averages, 512 acquired data points, excitation flip angle 25° and 10 rectangular NOE pulses with pulse duration of 10 ms, inter-pulse delay of 10 ms and excitation flip angle of 180°. The third baseline scan was run with identical parameters to the second with the exception of no excitation flip angle on the NOE pulses. The ratio of the resonance areas in the first and third scans were used to calculate correction factors for partial saturation, and in the same way the second and third baseline scans were used to calculate a NOE correction factor.

In the main experiment, a series of 512 individual ^31^P spectra were acquired throughout the exercise protocol described above with a temporal resolution of 5 s. The acquisition parameters were: TR 500 ms, TE 0.35 ms, bandwidth 2000 Hz, 10 averages, 512 acquired data points, excitation flip angle 25° and 10 rectangular NOE pulses with pulse duration of 10 ms, inter-pulse delay of 10 ms and excitation flip angle of 180°.

### Data processing

Spectra were processed using jMRUI version 2.2 [12] and quantified using a non-linear least squares algorithm (AMARES [13]). Resting ATP and total creatine concentrations were assumed to be 8.2 mM L^−1^ and 42 mM L^−1^ respectively: these commonly-used concentrations are based on extensive published values, and are reliable in healthy humans [14]. For muscle metabolite concentrations during exercise, reported values are the mean metabolite concentrations during the last minute of exercise bouts 2 and 3 (i.e. not including data from the ‘warm-up’ bout).

### Calculations

The myocellular free adenosine diphosphate (ADP) concentration was calculated making the standard assumption that that the creatine kinase reaction was at equilibrium, and using an equilibrium constant allowing for changes in pH [15].

The free energy available from ATP hydrolysis (ΔG'_ATP_, in kJ M^−1^) was calculated using:
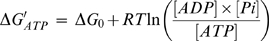
where ΔG_0_ is the standard free energy of ATP hydrolysis (−32.8 kJ M^−1^ [16]), R is the gas constant (8.31 J M^−1^ K^−1^) and T is temperature (310 K).

In the absence of large changes in pH, the halftime of PCr recovery after moderate exercise (PCr_t1/2_) was taken as an inverse index of mitochondrial function. PCr_t1/2_ was determined by fitting a monexponential equation, expressing PCr concentration as a function of time (*t*), of the form PCr (*t*) = 1 – exp (-*k t*), to data that had been normalized to resting values. Microsoft Solver was then used to establish the value of the rate-constant (*k*) that resulted in the smallest sum of the squared differences between actual and predicted PCr values, from which PCr_t1/2_  =  ln (2)/*k* [17]. The values reported are the means of the recovery halftimes after both exercise bouts.

The chemical shift of the phosphate (Pi) peak relative to phosphocreatine (PCr) (σ, in parts per million) was used to determine intracellular pH, according to the equation:
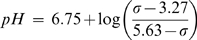



Muscle cross-sectional area was measured from ^1^H scout images (acquired as detailed above) using a freely available stereology software package (www.easymeasure.co.uk). The measured area comprised both the soleus and gastrocnemius muscles, as these are the active muscles during plantar flexion exercise [18].

### Statistical analysis

Statistical testing was carried out using SPSS 16.0 for Mac (SPSS Inc., Chicago, Illinois, USA). Shapiro-Wilk and Kolmogorov-Smirnov tests were used to establish whether data were normally distributed; no variable failed these tests of normality. Unpaired *t*-tests were used to test for differences between trekkers and climbers before the expedition. To quantify the significance of the effects of altitude exposure while controlling for potential differences between trekkers and climbers, a mixed analysis-of-variance was used; whether the subject was a trekker or a climber was coded as a between-subjects factor. There were, however, no significant between-subjects effects, thus the pre and post data are reported combined for brevity. Alpha was set at 0.05. All data are reported as mean ± S. E. M.

## Results

### Contrasts between trekkers and climbers at baseline

There was a significant difference in age between trekkers and climbers, with the climbers being 5 years older. In other respects the two groups were similar (Table 1).

**Table 1:** Subjects' descriptive data.

	Trekkers (n = 7)	Climbers (n = 7)	All (n = 14)
Age (years)	**31±2**	**38±3** *	35±2
Weight (kg)	74±2	83±6	78±3
Height (m)	1.80±0.02	1.80±0.01	1.80±0.01
BMI (kg m^−2^)	23±1	26±2	24±1
Calf muscle CSA (cm^2^)	4.5±0.3	4.8±0.2	4.6±0.2

*All numbers are means ± S.E.M.*

*different from trekkers at p<0.05.

**Figure 1:**
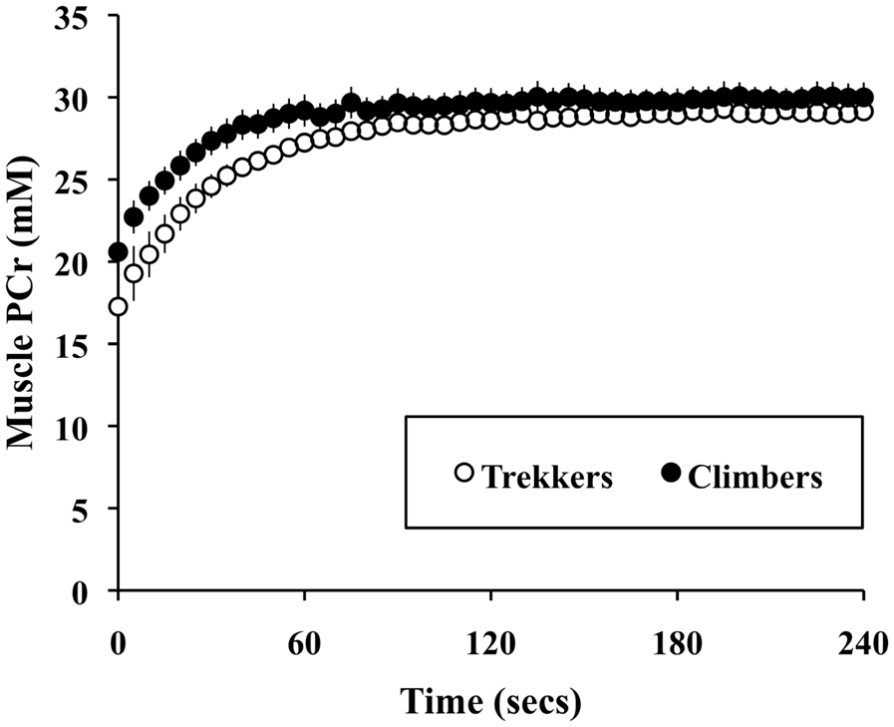
Muscle phosphocreatine kinetics in recovery from moderate exercise: differences between experienced climbers and altitude-naïve subjects. Data shown are means ± S.E.M.

At rest, the climbers had significantly higher concentrations of muscle cytosolic inorganic phosphate than the trekkers (climbers: 2.9±0.2 s vs. trekkers: 2.3±0.2, *n* = 14, *p*<0.05) (Table 2). Other resting phosphorus metabolites and resting muscle pH were similar between the groups. During and after recovery from exercise, there were no differences in phosphorus metabolites or pH (Table 2). However, there was an unexpected difference in PCr recovery halftimes (PCr_t1/2_) between trekkers and climbers before the expedition, with the trekkers having longer halftimes (trekkers: 22±2 s vs. climbers: 16±1 s, *n* = 14, *p*<0.01) (Figure 1).

**Table 2:** High-energy phosphates and pH in human skeletal muscle: trekkers vs. climbers before hypoxic exposure.

		Trekkers (n = 7)	Climbers (n = 7)
PCr (mM)	*Resting*	30.7±0.8	31.3±0.7
	*Exercising*	18.0±2.6	18.8±1.6
	*After recovery*	32.9±0.5	29.4±0.8
PCr_t1/2_ (s)	*During recovery*	**22.2±1.6**	**16.1±1.1** **
Pi (mM)	*Resting*	**2.3±0.2**	**2.9±0.2** *
	*Exercising*	11.6±1.8	12.9±2.2
	*After recovery*	1.3±0.2	1.5±0.1
ADP (µM)	*Resting*	28±3	26±2
	*Exercising*	53±5	54±8
	*After recovery*	20±2	24±2
ΔG'_ATP_ (mM J^−1^)	*Resting*	−63.3±0.5	−62.7±0.3
	*Exercising*	−58.0±0.7	−57.4±0.6
	*After recovery*	−65.7±0.4	−64.8±0.3
pH	*Resting*	7.09±0.005	7.10±0.003
	*Exercising*	6.94±0.05	7.05±0.02
	*After recovery*	7.08±0.02	7.08±0.01

*All values are means ± S.E.M.*

*different from trekkers at p<0.05;

**different from trekkers at p<0.01.

### The effect of altitude exposure on trekkers and climbers

**Figure 2:**
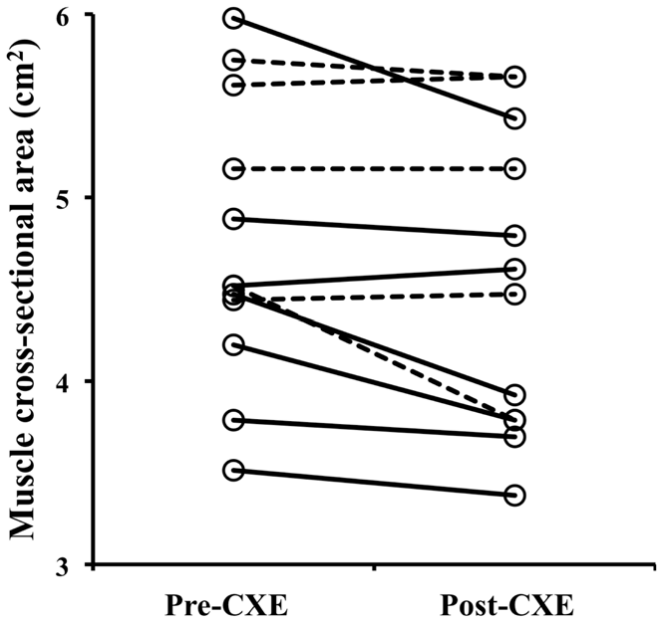
Individual changes in muscle cross-sectional area after a trip to high-altitude. Dashed lines are climbers, solid lines are altitude-naïve subjects. Change in mean values is significant at *p*<0.05, *n* = 12.

The combined subjects had a decreased calf muscle cross-sectional area following the expedition, from 4.7 to 4.5 cm^2^ (a loss of 4%, *p*<0.05) (Figure 2).

**Figure 3:**
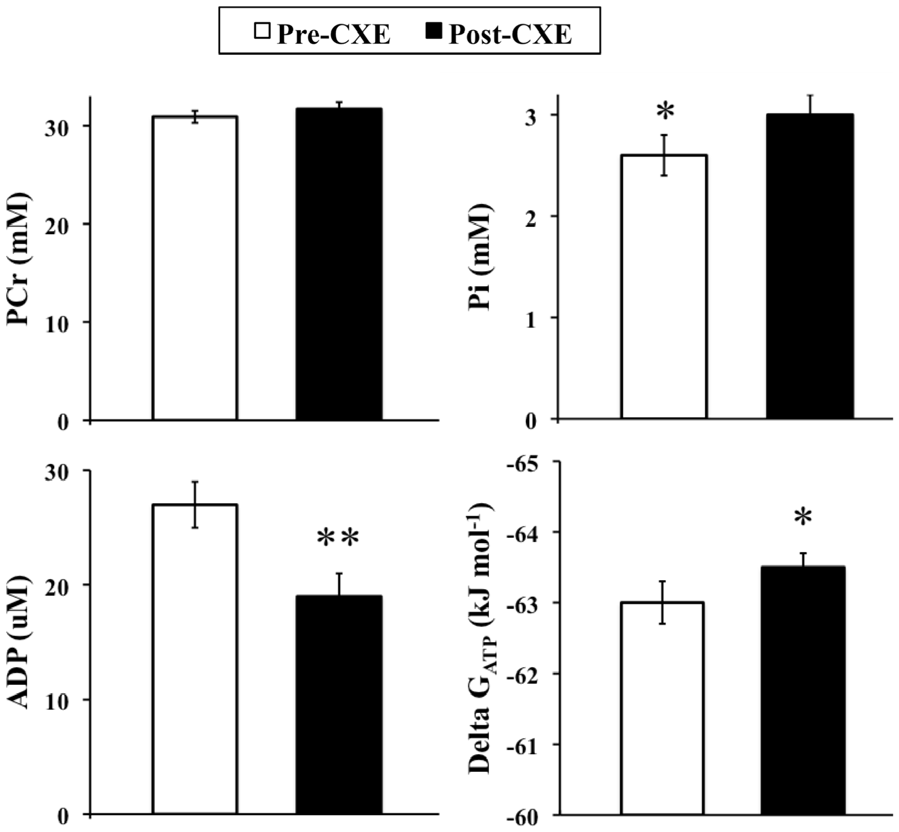
The effect of altitude exposure on resting high-energy phosphates in skeletal muscle. * different from pre-CXE at *p*<0.05; ** different from pre-CXE at *p*<0.01. CXE  =  Caudwell Xtreme Everest.

Resting muscle PCr concentration was not changed by exposure to high altitude. However, Table 3 shows that there were a number of significant effects of exposure on resting muscle phosphorus metabolites in the combined subjects. There was a 15% increase in inorganic phosphate (from 2.6 to 3.0 mM). Despite this, there was a 30% reduction in estimated resting ADP concentration, and an increase in the free energy available from ATP hydrolysis (Figure 3).

**Table 3:** The effect of altitude exposure on high-energy phosphates and pH in human skeletal muscle.

		PRE	POST
PCr (mM)	*Resting*	30.9±0.6	31.7±0.7
	*Exercising*	20.2±1.7	18.8±1.6
	*After recovery*	**32.9±0.5**	**29.4±0.8** **
PCr_t1/2_ (s)	*During recovery*	20±1	19±2
Pi (mM)	*Resting*	**2.6±0.2**	**3.0±0.2** *
	*Exercising*	11.6±1.8	12.9±2.2
	*After recovery*	1.3±0.2	1.5±0.1
ADP (µM)	*Resting*	**27±2**	**19±2** **
	*Exercising*	53±5	54±8
	*After recovery*	20±2	24±2
ΔG'_ATP_ (mM J^−1^)	*Resting*	**−63.0±0.3**	**−63.5±0.2** *
	*Exercising*	−58.0±0.7	−57.4±0.6
	*After recovery*	−65.7±0.4	−64.8±0.3
pH	*Resting*	**7.10±0.003**	**7.06±0.006** ***
	*Exercising*	6.99±0.03	7.00±0.02
	*After recovery*	**7.08±0.01**	**7.04±0.01** **

*Values are means ± SEM* (*n* = 12).

*different from PRE at p<0.05;

**different from PRE at p<0.01;

***different from PRE at p<0.001. There were no significant effects of trekker/climber grouping.

**Figure 4:**
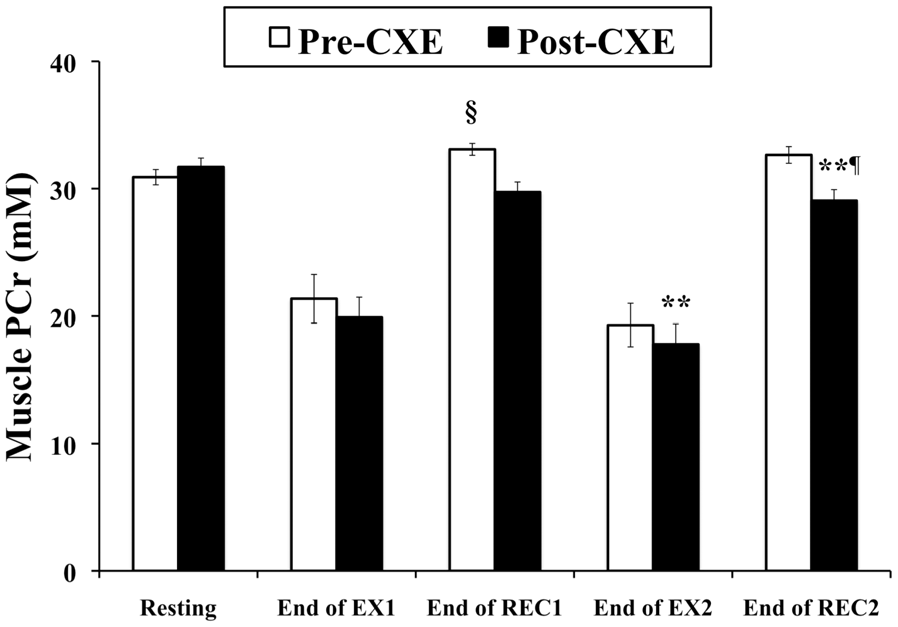
The effect of altitude exposure on phosphocreatine ‘overshoot’ in skeletal muscle. Values are means over the last minute of each period. CXE  =  Caudwell Xtreme Everest expedition, EX*x*  =  exercise bout *x*, REC*x*  =  recovery period after exercise bout *x*. ** different from pre-CXE at *p*<0.01; §End-REC1 PCr is different from resting PCr pre-CXE at *p*<0.05; ¶End-REC2 PCr is different from resting PCr post-CXE at *p*<0.05.

The concentrations of skeletal muscle high-energy phosphate metabolites during steady-state plantar-flexion exercise were unchanged in the combined group following exposure to altitude (Table 3); however, the mean concentration of PCr after full recovery from exercise was 11% lower following hypoxic exposure. This was due to both an absence of post-exercise phosphocreatine ‘overshoot’ and an exercise-induced loss of PCr (compared with resting values) after exposure (Figure 4).

There was no effect of altitude exposure on PCr recovery halftimes (PRE: 20±1 s vs. POST: 19±2 s, *n* = 12, *p*>0.05).

Resting muscle pH was significantly lower post-CXE (Table 3), and although end-exercise pH was not different post-CXE, muscle pH was still lower in the acclimatised state after recovery from exercise. In addition, there was a significant difference in post-exercise/recovery muscle pH compared with resting (pre-exercise) muscle pH post-exposure, which was not present in the pre-acclimatised state (Table 3).

## Discussion

The first significant finding of this study was an unexpected difference in PCr recovery halftime (and hence mitochondrial function) between climbers and trekkers at baseline, with the climbers having significantly shorter halftimes (and hence better mitochondrial function) than their altitude-naïve counterparts. The climbers also had significantly higher inorganic phosphate concentrations than trekkers (the possible significance of this difference will be discussed below). These results were particularly surprising considering the fact that none of the climbers had been to high altitude for at least 5 months. There was a significant difference in age between the groups, but this would be expected to result in the opposite effect, at least on mitochondrial function (the climbers were older, and mitochondrial function generally declines with age [19]). Nor did the climbers engage in any structured physical training prior to the expedition, being aware that differences between individuals in baseline cardiorespiratory fitness are unrelated to hypoxia tolerance (see [20] and references therein).

Several possible explanations suggest themselves. First, the climbers might have been different to the trekkers because years of climbing had selected against particular phenotypes (for example, those genetically ill-suited to high altitude hypoxia are unlikely to continually expose themselves to it). Second, altitude exposure might induce stable changes in phenotype, perhaps through epigenetic modifications. Finally, it may be that repeated hypoxic exposure causes more conventional physiological adaptations that are unusually persistent. There are no published data on the timecourse of ‘de-acclimatisation’ after hypoxic exposure. The most relevant information therefore probably comes from experiments examining the timecourse of detraining after stopping prolonged exercise training. Coyle et al. observed that subjects who had previously been well-trained but who had stopped training altogether for 84 days still had, on average, a 17% higher 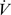
O_2_max than controls who had never trained at all [21]. This was due to persistent peripheral adaptations (larger mixed-arterial/venous O_2_ difference, and therefore better O_2_ extraction by skeletal muscle). So despite the fact that none of the climbers had climbed for at least 5 months prior to this study, it seems possible that the difference in mitochondrial function observed was a long-term (or even stable and transmissible) effect of exposure to high-altitude hypoxia. More work investigating these differences is clearly warranted, as are comparisons with high-altitude natives and the offspring of successful climbers.

Exposure to hypobaric hypoxia is associated with an involuntary loss of body mass [22], whether under laboratory conditions [23], [24], [25] or in the field [26], [27]. In women, nitrogen balance is negative soon after exposure to 4300 m altitude [28] and remained negative in men throughout a 7,102 m ascent [29]. In keeping with these observations, we observed a significant reduction in muscle cross-sectional area after hypoxic exposure (Figure 2). There was no difference in the degree of atrophy between the groups (no statistically significant effect of trekker/climber grouping).

Such weight loss does not seem to relate simply to excessive metabolic demands related to exertion. Indeed, physical activity levels (PAL, assessed as maximal exertional metabolic rate as a multiple of basal metabolic rate (BMR)) are normally 2.2–2.5 at sea level, and twice that in trained athletes. However, near Everest's summit, PAL is limited to 2.0–2.7, meaning that exertional energy loss is minimized [30]. Thus, although perceived exertion is great, actual energy expenditure is much less [31], [32]. The notion that weight loss is not purely due to excessive metabolic demands is given further credence by the observation that obese subjects exercising three times each week in 15% oxygen lose more weight than those exercising in air [33].

A variety of factors are thought to contribute to altitude-induced weight loss. First, energy expenditure may rise at altitude, due in part to an increase in BMR [34], [35], [36], [37]. This effect may be altitude-dependent: BMR has risen by 6% in men at 3,650 m [38], by 10% at 3,800 m [37], and by 27–28% in men and women by day 2–3 at 4,300 m [32], [36]. In the absence of calorie supplementation, such absolute increases in BMR do not seem sustained [36], [37], [39], [40], although BMR per unit mass may actually remain elevated [38], [41].

Second, pro-inflammatory cytokines may play a role. In eight sea-level residents, the interleukin-6 (IL-6) response to 60 min of bicycle ergometer exercise was found to be greater during exposure to acute hypoxia (4100 m altitude) than that seen in normoxia [42]. After 6 weeks of exposure to 4100 m, IL6 levels remain elevated [42], a finding in keeping with similar observations over four days of exposure to 4350 m in males [43] and with 12 days of exposure to 4300 m in women [44]. Interleukin-6 is implicated in the pathogenesis of cancer-associated weight loss, driving lipid catabolism and muscle protein catabolism [45], [46], perhaps through both lysosomal (cathepsin) and non-lysosomal (proteasome) pathways [47].

There was no decline in volume-scaled mitochondrial function (PCr_t1/2_) after hypoxic exposure (Table 3). Taken in the context of significant atrophy, this means that whole muscle aerobic capacity was reduced. Yet, rather surprisingly in the face of significant muscle atrophy and a loss of aerobic capacity, the expedition did not have any adverse effects on muscle function during exercise. Subjects were able to complete the same exercise tasks pre and post exposure, and exercising metabolites were unchanged (Table 3). Although at variance with previous reports that muscle mitochondrial enzyme activities (per unit of cross sectional area) are decreased by hypoxic exposure [4], our results suggest that *in vivo* function might somehow be maintained. Further experiments specifically targeted at illuminating changes in muscle mitochondrial function *in vivo* in response to hypoxic exposure are required.

When fully recovered after a period of exercise, PCr concentrations are often higher than pre-exercise values, a phenomenon known as PCr ‘overshoot’ [48]. There is very little published literature regarding the mechanisms underlying PCr overshoot in skeletal muscle. One hypothesis states that PCr overshoot is the result of a slow decay in one of the signals that directly activates oxidative phosphorylation [49]. If this were the case, then the data here suggest a tightening of off-exercise oxygen kinetics, perhaps to prevent unnecessary oxygen consumption. An alternative explanation would be that inorganic phosphate is being lost during exercise (perhaps due to calcium-phosphate precipitation [50]).

There were several changes in resting muscle high-energy phosphates after hypoxic exposure. While the changes in estimated free [ADP] will be discussed below, the increase in muscle phosphate is noteworthy because it mirrors a difference that was observed between the climbers and trekkers at baseline. There are two possible mechanisms for an increased steady-state cell [Pi]. The first is an increased Na^+^-dependent Pi uptake. This mechanism is poorly understood, but can be driven by an increase in insulin, possibly indirectly via effects on the Na^+^ gradient [51]. The second theoretical possibility is reduced permeability to Pi efflux, although convincing examples are currently lacking.

Our calculations of [ADP] rest on a number of assumptions regarding muscle metabolite (ATP and creatine) concentrations. Although these assumptions are generally sound [14], one cannot discount the possibility that the extreme conditions experienced by our subjects invalidated them. Thus our findings need to be interpreted with caution. PCr concentration was calculated from the PCr/ATP ratio (assuming an intramuscular [ATP] of 8.2 mM L^−1^) and was unchanged by the expedition, strongly suggesting that [ATP] was also unchanged. However, we did not directly measure creatine and the observed increase in calculated [ADP] could be accounted for by an increase in total creatine. Despite these reservations, it seems reasonable that [ADP] might have decreased in response to hypoxia. It is now widely accepted that mitochondrial oxidative rate is matched to ATP demand by feedback mediated by intracellular phosphorylation potential or some function of it. Therefore a reduction in the resting ADP concentration would indicate either a change in the control parameters linking oxidative rate to [ADP] or a reduction in resting muscle oxidative rate.

Resting (but not exercising) pH was significantly lower following exposure. This was not a result of systemic ketoacidosis, as there was no correlation between pH and β-hydroxybutyrate (correlation not shown). We therefore suggest that it was the result of either increased metabolic proton production or decreased capacity for cellular proton extrusion (for example, a reduced activity or sensitivity of Na^+^/H^+^-ATPase).

### Limitations

There are a number of limitations to this study. First, the trekkers and climbers were different from each other before the study began and we have chosen to treat the subjects as a single group (as well as separately). We justify this based on an absence of any statistical evidence that the groups responded differently. Second, several of the climbers were very slow descending from altitude before revisiting Oxford. However, this has provided an unexpected benefit: because there were no differences between the responses of trekkers (who returned to Oxford immediately) and climbers, it is unlikely that the observed changes were acute responses. For example, this shows that the post-exposure reduction in muscle pH (equally present in both groups) was not due to an acute disturbance in acid-base status.

### Conclusions

We used magnetic resonance spectroscopy and imaging to study the effects of a trip to high altitude (Mount Everest) on a mixed cohort of altitude-naïve trekkers and experienced climbers. The climbers had unexpectedly better mitochondrial function than the trekkers at baseline. Both groups responded similarly to the hypoxic insult. Climbers had higher resting [Pi] than trekkers before the expedition and resting [Pi] was raised across both groups on their return. There was significant muscle atrophy post-CXE, yet exercising metabolites were unchanged. These results suggest that, in response to high altitude hypoxia, skeletal muscle function is maintained in humans, despite significant atrophy.
